# Role of autophagy in modulating post-maturation aging of mouse oocytes

**DOI:** 10.1038/s41419-018-0368-5

**Published:** 2018-02-22

**Authors:** Fei-Hu Lin, Wei-Ling Zhang, Hong Li, Xiao-Dan Tian, Jie Zhang, Xiao Li, Chuan-Yong Li, Jing-He Tan

**Affiliations:** Shandong Provincial Key Laboratory of Animal Biotechnology and Disease Control and Prevention, College of Animal Science and Veterinary Medicine, Shandong Agricultural University, Tai’an City, 271018 P. R. China

## Abstract

Mechanisms for post-maturation oocyte aging (PMOA) are not fully understood, and whether autophagy plays any role in PMOA is unknown. To explore the role of autophagy in PMOA, expression of autophagosomes and effects of the autophagy (macro-autophagy) activity on PMOA were observed in mouse oocytes. Oocyte activation rates and active caspase-3 levels increased continuously from 0 to 18 h of in vitro aging. While levels of microtubule-associated protein light chain 3 (LC3)-II increased up to 12 h and decreased thereafter, contents of p62 decreased from 0 to 12 h and then elevated to basal level by 18 h. However, the LC3-II/I ratio remained unchanged following aging in different media or for different times. During in vitro aging up to 12 h, upregulating autophagy with rapamycin or lithium chloride decreased activation susceptibility, cytoplasmic calcium, p62 contents, oxidative stress, caspase-3 activation and cytoplasmic fragmentation while increasing developmental competence, LC3-II contents, LC3-II/I ratio, mitochondrial membrane potential, spindle/chromosome integrity and normal cortical granule distribution. Downregulating autophagy with 3-methyladenine (3-MA) produced opposite effects on all these parameters except cytoplasmic fragmentation. After 12 h of aging culture, however, regulating autophagy with either rapamycin/lithium chloride or 3-MA had no impact on oocyte activation susceptibility. It is concluded that autophagy plays an important role in regulating PMOA. Thus, during the early stage of PMOA, autophagy increases as an adaptive response to prevent further apoptosis, but by the late stage of PMOA, the activation of more caspases blocks the autophagic process leading to severer apoptosis.

## Introduction

If not fertilized or activated in time, mature oocytes undergo a time-dependent process of post-maturation oocyte aging (PMOA)^1–6^. The term PMOA is used instead of post-ovulatory oocyte aging because it covers both in vivo and in vitro matured oocytes. Although it is known that PMOA has marked detrimental effects on embryo development^5,7–9^ and offspring^10,11^, the mechanisms for PMOA are not fully understood. Studies have shown that PMOA leads to apoptosis. For example, the expression of the antiapoptotic protein BCL2 was gradually reduced during postovulatory oocyte aging^12–14^. Fertilization-like Ca^2+^ responses induced by injection of sperm cytosolic factor triggered cell death, rather than activation, in aged oocytes^15^. Furthermore, the aged oocytes exhibited extensive cytoplasmic and DNA fragmentation and activation of protein caspases^15,16^.

Autophagy is an evolutionarily conserved mechanism by which the cytoplasmic contents are sequestered within autophagosomes and delivered to the lysosome for degradation^17^. Autophagy is recognized as a cell survival mechanism in starving cells, and it also functions in cell death^18^. Autophagy has been observed in mouse^19^ and porcine oocytes^20–22^. Furthermore, in a study to visualize the different routes of ovarian oocyte elimination in rats, Escobar Sánchez et al.^23^ demonstrated that all phases of the estrous cycle contained dying oocytes that tested positive simultaneously for apoptosis and autophagy markers, suggesting that the proteins involved in both the apoptosis and autophagy processes are present in the same cell at the same time. We thus hypothesize that autophagy may play a role in regulating PMOA. However, there are no reports on the role of autophagy in regulating PMOA.

The objective of this study was to explore the role of autophagy in modulating PMOA by observing expression of autophagosomes in mouse oocytes aging for different times and by determining the effects of autophagic activities on the manifestations of aging oocytes.

## Results

### Activation rates and autophagosome levels in oocytes aging in different media for different times

**Fig. 1:**
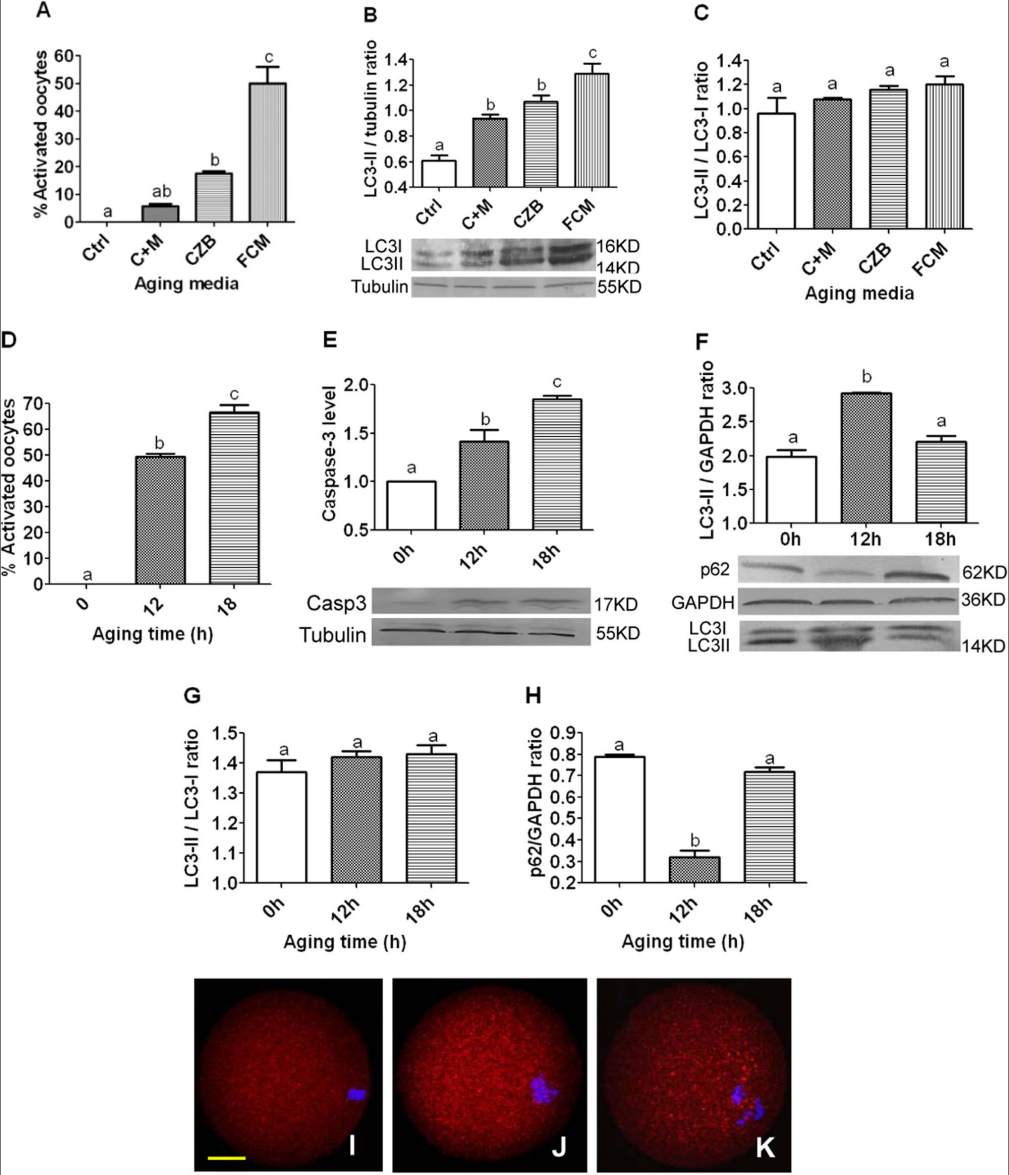
Activation rates and levels of LC3, P62 and active caspase-3 after mouse DOs were aged for different times in different media. Graphs **a** and **d** show percentages of activated oocytes. While **a** shows freshly collected control (Ctrl) oocytes and oocytes aging for 12 h in CZB + MG132 (C + M), CZB or FCM, **d** shows oocytes aging for different times in FCM. Each treatment was repeated 3–4 times with each replicate containing about 30 oocytes. Graphs **b** and **c** show LC3-II quantification by western blotting in oocytes aging for 12 h in different media. While **b** shows LC3-II contents (LC3-II/tubulin ratio), **c** shows the LC3-II/LC3-I ratio. Each treatment was repeated three times with each replicate containing about 200–250 oocytes. Graph **e** shows relative levels of active caspase-3 in oocytes aging in FCM for different times as detected by western blotting. Each treatment was repeated three times with each replicate containing about 300-400 oocytes. Graphs **f**, **g** and **h** show LC3-II contents (LC3-II/GAPDH ratio), LC3-II/LC3-I ratio and p62 (p62/GAPDH ratio), respectively, in oocytes aging in FCM for different times. Each treatment was repeated three times with each replicate containing about 200–250 oocytes. **a**–**d** Values with a different letter above bars differ significantly (*P* < 0.05). Micrographs **i**, **j** and **k** are confocal images showing autophagosome (LC3-II puncta) distribution in oocytes aging in FCM for 0, 12 and 18 h, respectively. These are the merged pictures with chromosomes and LC3-II puncta pseudo colored blue and red, respectively. The bar is 15 µm and applies to all images

To observe the relationship between activation susceptibility and autophagy activity, newly ovulated oocytes collected at 13 h after hCG injections were aged for 12 h in different media before ethanol treatment for activation or western blotting for measurement of LC3 concentrations. While the highest activation rate was observed in oocytes aging in the FasL-rich conditioned medium (FCM), the lowest was in the newly ovulated control oocytes and in oocytes aging in CZB medium supplemented with MG132, with that in oocytes aging in CZB alone in between (Fig. 1a). The highest level of LC3-II was also detected in oocytes aging in FCM, the lowest was in control oocytes, with that in oocytes aging in CZB alone and in CZB + MG132 in between (Fig. 1b). However, the ratio of LC3-II/LC3-I did not change significantly after different treatments (Fig. 1c). Taken together, during the first 12 h of in vitro aging, the autophagosome contents of mouse oocytes increased with increasing activation susceptibility although changes in the rate of autophagic flux were not obvious, suggesting that autophagic activities increased in aging oocytes through both enhanced synthesis and conversion of LC3-I to LC3-II.

The high susceptibility to activating stimulus as an early manifestation of oocyte apoptosis seemed to contradict with the high autophagic activities observed in oocytes aging in FCM for 12 h, as it is generally accepted that autophagy blocks the induction of apoptosis^24^. Because it has been reported that the apoptosis-associated activation of caspases can switch off the autophagic process^24^, we hypothesized that the increase in autophagy observed at 12 h of oocyte aging might represent a stress response to prevent further apoptosis, but after that, autophagy would decrease as more caspases were activated with further oocyte aging. To test the hypothesis, freshly ovulated oocytes were aged in FCM medium for different times before ethanol activation or western blot analysis. Although both activation rate (Fig. 1d) and active caspase-3 level (Fig. 1e) increased, the level of LC3-II (Fig. 1f) decreased significantly from 12 h to 18 h of in vitro aging. The LC3-II/LC3-I ratio, however, did not change significantly among oocytes aging for different times (Fig. 1g). The level of p62 decreased significantly from 0 to 12 h of in vitro aging but by 18 h of culture, it increased to the same level as observed in 0 h oocytes (Fig. 1h). This dynamics of p62 protein further confirmed a high autophagic activity at 12 h and a decrease in autophagic activity at 18 h of in vitro aging, as it is known that the expression level of p62 usually correlate inversely with autophagic activity. For example, upon starvation of mouse embryonic fibroblasts and HepG2 cells, p62 was initially degraded by autophagy, but was restored back to basal levels during prolonged starvation^25^. Furthermore, our immunofluorescence microscopy also indicated that the level of autophagosomes (LC3-II puncta) increased at 12 h but decreased significantly at 18 h of in vitro aging (Fig. 1i, j, k). Taken together, the results showed that oocyte aging is closely correlated with the dynamics of autophagy. Thus, during the early stage of oocyte aging, autophagy increases as an attempt to delay further apoptosis, but by late stages of aging, the activation of more caspases blocks the autophagic process.

### Effects of regulating autophagic activities on activation susceptibility, cytoplasmic calcium levels and developmental potential of aging oocytes

**Fig. 2:**
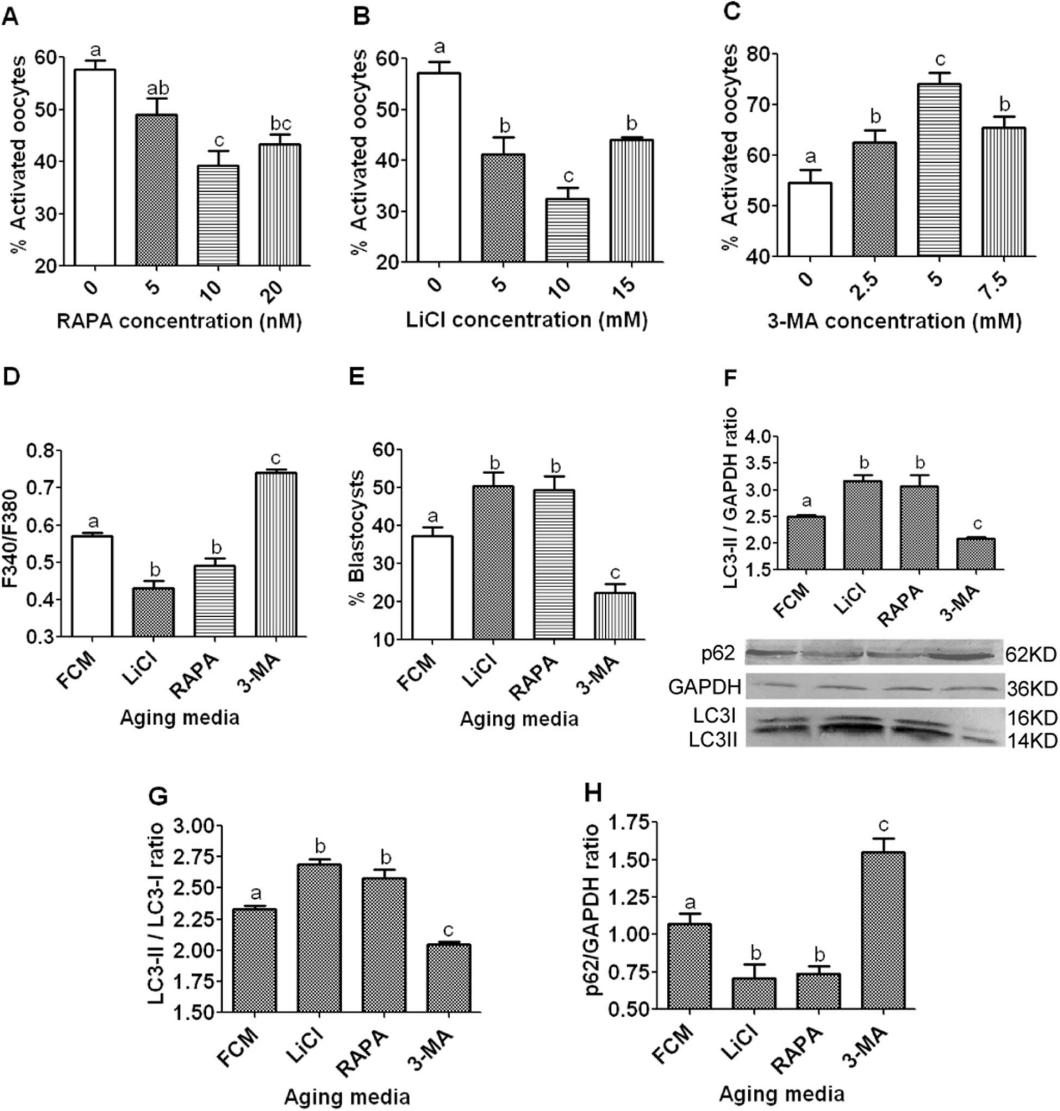
Effects of regulating autophagy activity on activation rates (**a**–**c**), cytoplasmic calcium concentrations (**d**), blastocyst percentages (**e**), and levels of and LC3 (**f** and **g**) or p62 (**h**) expression in aging mouse oocytes . In graphs **a**, **b** and **c**, newly ovulated oocytes collected 13 h post hCG were cultured for 12 h in FCM containing different concentrations of rapamycin (RAPA), 3-MA or lithium chloride (LiCl). In graphs **d**–**h**, newly ovulated oocytes were aged for 12 h in FCM alone or in FCM containing 10-nM RAPA, 5-mM 3-MA or 10 mM LiCl before examination. For oocyte activation, each treatment was repeated 3–4 times with each replicate containing about 30 oocytes. Calcium levels (the ratio of F340/380) was measured using the Ca^2+^-sensitive dye Fura-2 AM. The signals were collected for 20 min during in vitro culture of aged oocytes with ionomycin added at 5 min. Each treatment was repeated three times with each replicate containing about 20 oocytes. Blastocyst percentages were calculated from four-cell embryos from activated oocytes and the percentages of four-cell embryos did not differ between treatments. Levels of LC3-II (LC3-II/GAPDH ratio) and p62 (p62/GAPDH ratio) expression was determined by western blotting, and each treatment was repeated three times with each replicate containing about 200–250 oocytes. **a**–**c** Values with a different letter above bars differ significantly (*P* < 0.05)

To up or down regulate autophagic activities, newly ovulated oocytes were aged for 12 h in FCM containing various concentrations of rapamycin, lithium chloride (LiCl), or 3-methyladenine (3-MA). Control oocytes were aged in FCM with neither rapamycin, LiCl, nor 3-MA. At the end of the aging culture, oocytes were examined for activation rates, cytoplasmic calcium levels, blastocyst rates and LC3 and p62 concentrations. When oocytes were aged with optimal concentrations of rapamycin (10 nM) or LiCl (10 mM), while activation rates (Fig. 2a,b), cytoplasmic calcium levels (Fig. 2d) and p62 contents (Fig. 2h) decreased, blastocyst rates (Fig. 2e), LC3-II levels (Fig. 2f) and LC3-II/I ratio (Fig. 2g) increased significantly, compared to those in control oocytes cultured in FCM alone. When oocytes were aged with 3-MA (5 mM), however, while activation rates (Fig. 2c), cytoplasmic calcium levels (Fig. 2d) and p62 contents (Fig. 2h) increased, blastocyst rates (Fig. 2e), LC3-II levels (Fig. 2f) and LC3-II/I ratio (Fig. 2g) decreased significantly, compared to those in control oocytes. Furthermore, our correlation analysis revealed a significant negative correlation of LC3-II levels with activation rates (*r* = −0.998) but a positive correlation with the blastocyst rates (*r* = 0.988) in oocytes aging in different media.

### Effects of regulating autophagic activities on oxidative stress and apoptosis of aging oocytes

**Fig. 3:**
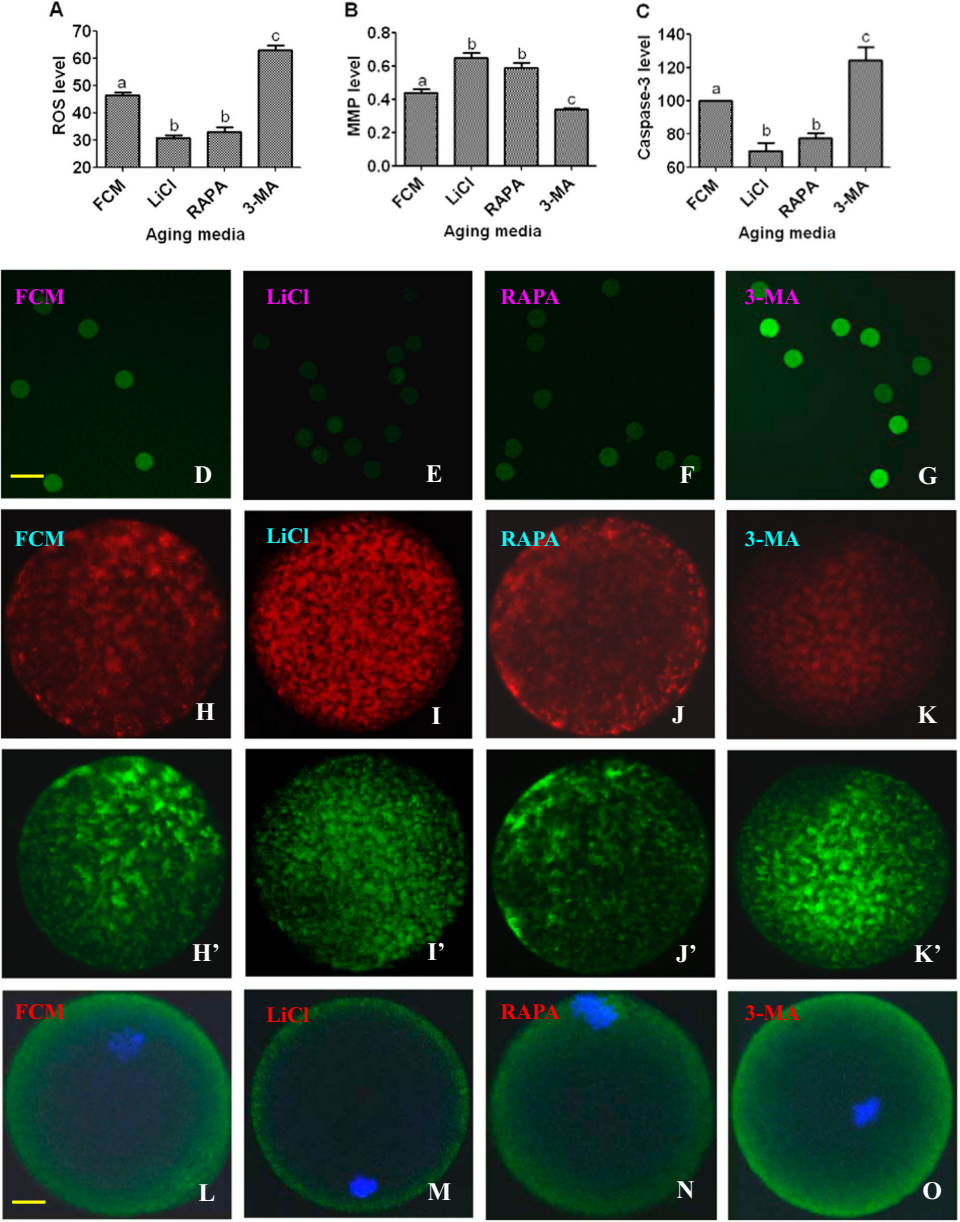
Effects of regulating autophagy activity on levels of ROS, mitochondrial membrane potential (MMP) and active caspase-3 of aging mouse oocytes. Freshly collected oocytes were aged for 12 h in FCM alone or in FCM containing 10-nM RAPA, 10-mM LiCl or 5-mM 3-MA before examination. Graph **a** shows relative levels of ROS (fluorescence intensity value, FIV). Graph **b** shows MMP (red/green fluorescence intensity) as determined by staining with MMP-specific probe JC-1. Graph **c** shows relative levels of active caspase-3 as determined by immunofluorescence; the caspase-3 value of oocytes aging in FCM alone was set as 100 and the other values were expressed relative to this value. Each treatment was repeated three times with each replicate containing about 20 oocytes. **a**–**c** Values with a different letter above bars differ significantly (*P* < 0.05). **d**, **e**, **f** and **g** are confocal images showing the ROS FIV in oocytes aging in different media. **H**/**H**’, **I**/**I**’, **J**/**J**’ and **K**/**K**’ are confocal images showing JC-1 staining intensity in oocytes aging in different media. **H** and **H**′, **I** and **I**′, **J** and **J**′, and **K** and **K**′ are the same oocytes observed in TRITC channel (red fluorescence) and in FITC channel (green fluorescence), respectively. **L**, **M**, **N** and **O** are confocal images showing levels of active caspase-3 in oocytes aging in different media. DNA and caspase-3 are colored blue and green, respectively. Bar is 120 µm in images **D**–**G** and is 12 µm in images **H**–**O**

Freshly ovulated mouse oocytes were aged for 12 h in FCM containing rapamycin, LiCl or 3-MA. Control oocytes were aged in FCM with neither rapamycin, LiCl nor 3-MA. At the end of the aging culture, oocytes were examined for levels of ROS, mitochondrial membrane potential (MMP) and active caspase-3. The results showed that compared to those in control oocytes aging in FCM alone, levels of ROS and active caspase-3 decreased in oocytes aging with rapamycin or LiCl but increased significantly in oocytes aging with 3-MA (Fig. 3). In contrast, while the level of MMP increased in oocytes aging with rapamycin or LiCl, it decreased significantly in oocytes aging with 3-MA, compared to that in control oocytes.

### Effects of regulating autophagic activities on spindle/chromosome morphology and cortical granules (CGs) distribution of aging oocytes

**Fig. 4:**
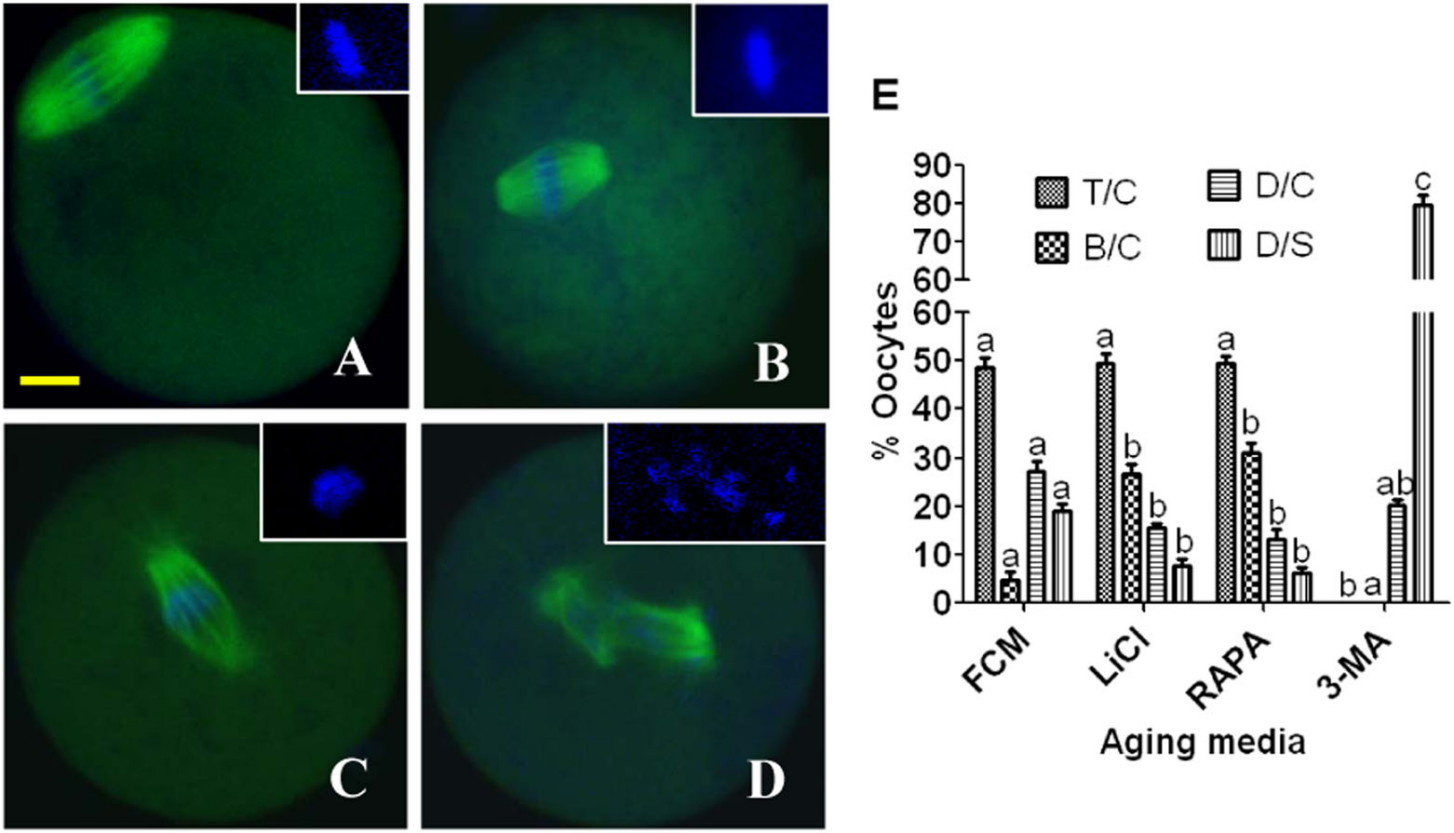
Effects of regulating autophagy activity on spindle/chromosome morphology of aging mouse oocytes. Oocytes that had aged for 12 h in FCM or FCM containing 10 nM Rapamycin, 10 mM LiCl or 5 mM 3-MA were cultured for 24 h in CZB medium before examination for morphology of spindles and chromosomes. In the confocal images, DNA and α-tubulin were pseudo-colored blue and green, respectively. Bar is 10 µm and applies to all images. Image **a** shows an oocyte with a tine-pole spindle and chromosomes congressed on the metaphase plate (T/C), image **b** shows an oocyte with a barrel-shaped spindle and congressed chromosomes (B/C), image **c** shows an oocyte with a disintegrated spindle and congressed chromosomes (D/C), and image **d** shows an oocyte with a disintegrated spindle and scattered chromosomes (D/S). Graph **e** shows percentages of oocytes with different spindle/chromosome configurations following oocytes were aged in different media. Each treatment was repeated 3 times with each replicate containing about 20 oocytes. **a**–**c** Values with different letters above bars differ significantly (*P* < 0.05) within spindle/chromosome morphologies

Oocytes that had aged for 12 h in FCM alone or in FCM containing 5 mM 3-MA, 10 nM rapamycin or 10 mM LiCl were cultured for 24 h in CZB medium before examination for spindle/chromosome morphology or CGs distribution. Four types of spindle/chromosome morphology were observed: tine pole (Fig. 4a) or barrel-shaped spindles (Fig. 4b) with chromosomes congressed on the metaphase plate, and disintegrated spindles with congressed (Fig. 4c) or scattered chromosomes (Fig. 4d). Compared to those in control oocytes aging in FCM alone, the presence of rapamycin or LiCl in aging medium significantly increased the proportion of oocytes with barrel-shaped spindles and congressed chromosomes while decreasing the oocytes with disintegrated spindles and congressed or scattered chromosomes (Fig. 4e). The presence of 3-MA, however, significantly decreased the number of oocytes with tine pole or barrel-shaped spindles while significantly increasing the percentage of oocytes with disintegrated spindles and scattered chromosomes.

**Fig. 5:**
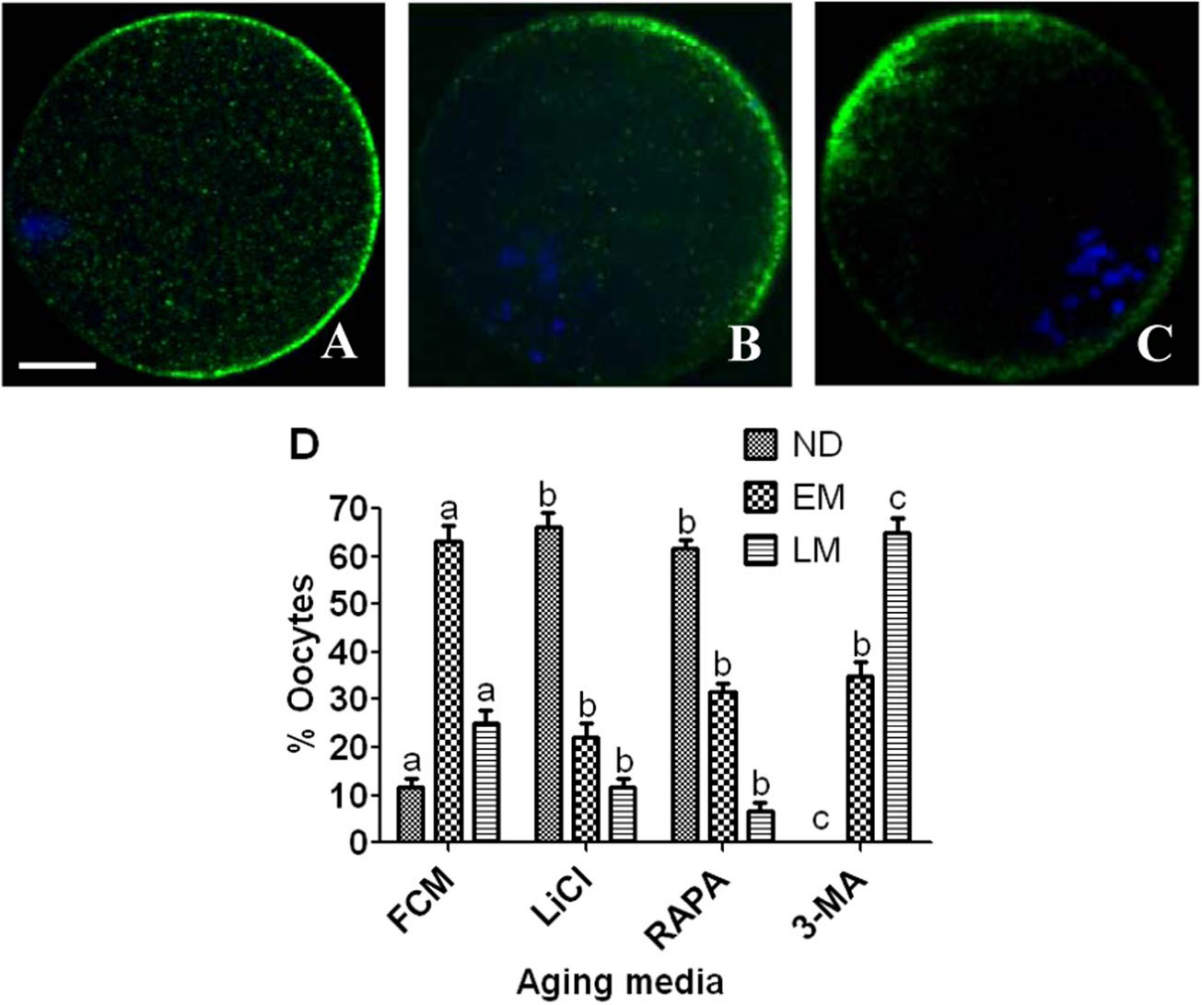
Effects of regulating autophagy activity on CGs distribution of aging mouse oocytes. Oocytes that had aged for 12 h in FCM or FCM containing 10 nM rapamycin, 10 mM LiCl or 5 mM 3-MA were cultured for 24 h in CZB medium before examination for distribution of CGs. In the confocal images, DNA and CGs were pseudo-colored blue and green, respectively. Bar is 15 µm and applies to all images. Images **a**, **b** and **c** show oocytes with normal distribution (ND), early migration (EM) and late migration (LM) of CGs, respectively. Graph **d** shows percentages of oocytes with different patterns of CGs distribution following oocyte aging in different aging media. Each treatment was repeated three times with each replicate containing about 20 oocytes. **a**–**c** Values with different letters above bars differ significantly (*P* < 0.05) within patterns of CGs distribution

Three patterns of CGs distribution were observed: (a) normal distribution (ND) with all the CGs tidily aligned beneath the plasma membrane except for the CG-free domain above the spindle (Fig. 5a); (b) early migration (EM) of CGs inward and toward the vegetal pole (Fig. 5b); and (c) late migration (LM) of CGs further inward and toward the vegetal pole (Fig. 5c). Over 60% of the oocytes showed EM, ND and LM patterns of CGs distribution following aging in FCM alone, with rapamycin/LiCl or with 3-MA, respectively (Fig. 5d). Taken together, the results suggested that autophagy played an important role in maintaining chromosome/spindle integrity and normal CGs distribution during PMOA.

### Effects of regulating autophagic activities on cytoplasmic fragmentation of aging oocytes

**Fig. 6:**
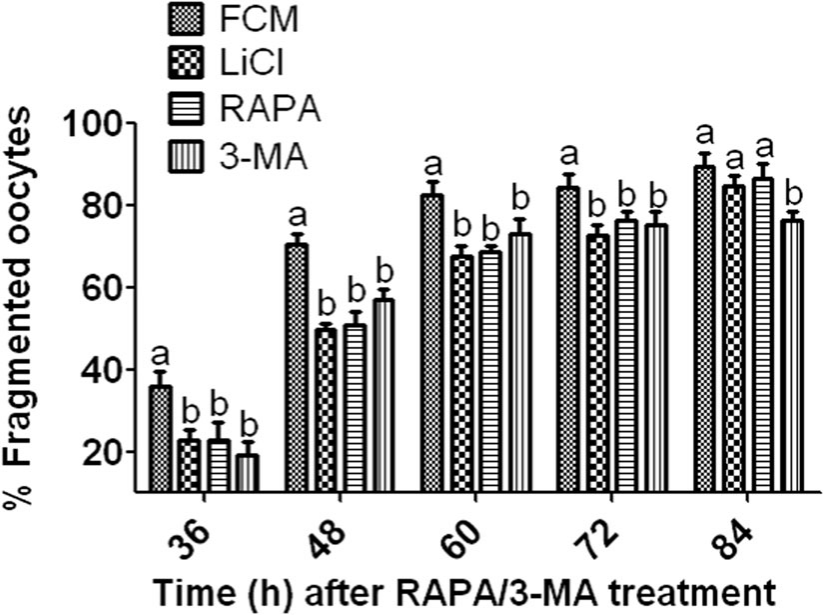
Fragmentation rates at different times after newly ovulated oocytes were aged for 12 h in FCM with or without 10 nM RAPA, 10 mM LiCl or 5 mM 3-MA. Each treatment was repeated four times with each replicate containing about 30 oocytes. a, b: Values with different letters above bars differ significantly (*P* < 0.05) within time points

Newly ovulated oocytes were aged for 12 h in FCM with or without rapamycin, LiCl or 3-MA, and at the end of the aging culture, the oocytes were incubated in regular CZB medium for different times before observation for cytoplasmic fragmentation. Oocytes with a clear moderately granulate cytoplasm, and an intact first polar body, were considered to be un-fragmented, and oocytes with more than two asymmetric cells were considered to be fragmented^26^. As expected, supplementation of rapamycin or LiCl to aging culture medium significantly reduced cytoplasmic fragmentation of aging oocytes (Fig. 6). Unexpectedly, however, treatment with 3-MA did not enhance but rather inhibited cytoplasmic fragmentation of aging oocytes. The results suggested that autophagy is involved in the regulation of cytoplasmic fragmentation of aging oocytes but the unexpected effect of 3-MA on cytoplasmic fragmentation of aging oocytes needs further investigations.

### Regulating autophagy after 12 h of aging culture had no effects on oocyte activation susceptibility

**Fig. 7:**
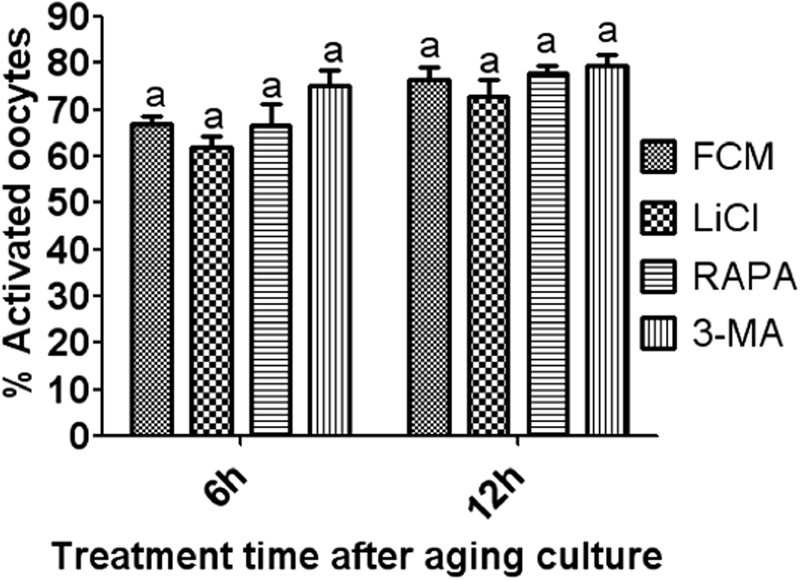
Activation rates after mouse oocytes were treated with rapamycin, LiCl or 3-MA for 6 or 12 h following a regular aging culture in FCM for 12 h . Each treatment was repeated 3–4 times with each replicate containing about 30 oocytes. a: Values with a common letter above bars do not differ significantly (*P* > 0.05)

To further confirm the critical role of autophagy in modulating oocyte aging, effects of regulating autophagy at the time when the autophagosome level had significantly dropped on oocyte activation susceptibility were observed. Following a regular aging culture in FCM alone for 12 h, oocytes were treated with rapamycin, LiCl or 3-MA for 6 or 12 h before observation for ethanol activation rates. The results showed that after in vitro aging for 12 h, treatment with either rapamycin, LiCl or 3-MA for either 6 or 12 h did not affect oocyte activation rates (Fig. 7), suggesting that up or down regulating autophagy at the time when the autophagosome level had significantly declined had no effects on oocyte activation susceptibility.

## Discussion

In this study, oocyte aging in different media for 12 h or in FCM for different times showed that LC3-II contents, but not LC3-II/I ratio, were correlated with oocyte aging parameters, and that the expression level of p62 correlated inversely with LC3-II contents but not with LC3-II/I ratio. It is known that during autophagy induction, LC3-I is lipidated to LC3-II, leading to an increased LC3-II/I ratio. However, while the LC3-II/I ratio is often used as a marker for autophagy in various tissues, there are reports that the LC3-II amount is more representative of autophagy activation than the LC3-II/I ratio is^27^. The p62 protein has been used as an index of autophagic degradation. Thus, while inhibition of autophagy correlates with increased levels of p62, decreased p62 levels are associated with autophagy activation^28^. Taken together, the present results suggest that it is the LC3-II amount but not the LC3-II/I ratio that reflects the level of autophagic activity in aging oocytes. Furthermore, the synthesis of p62 in somatic cells depends on its transcriptional upregulation^25^. Because transcription is inhibited in mature oocytes, the translation of maternal mRNAs stored in the cytoplasm during oogenesis^29^ may be the primary source for LC3 and p62 expression in aging oocytes.

In this study, while rapamycin and LiCl were used to upregulate, 3-MA was used to down regulate autophagic activities in aging oocytes. LiCl was used to set up an mTOR-independent control for rapamycin, as it has been reported that lithium induced autophagy independently of mTOR by inhibiting inositol monophosphatase^30^. The results showed that activating autophagy with LiCl produced similar effects as with rapamycin on oocyte aging parameters. This suggests that autophagy in aging oocytes can be activated through alternative pathways other than the mTOR signaling.

The present results suggested that during the early stage of PMOA, autophagy increased as a stress response to prevent further apoptosis; then, the activated autophagy promoted caspase activation; and by the late stage of PMOA, the activation of more caspases in turn blocked the autophagic process itself. In somatic cells, while autophagy may act as a cell survival process by acting as a stress response, delaying caspase activation, and removing damaged organelles, it can also lead to apoptosis by enhancing caspase activation^31^ or by inhibiting expression of the pro-survival gene Bcl-2^32^. Furthermore, although autophagy blocks the induction of apoptosis in general, apoptosis-associated caspase activation shuts off the autophagic process^24^. For example, apoptosis induced by the proapoptotic protein Bax reduced autophagy by enhancing caspase-mediated cleavage of Beclin 1 at D149^33^.

Both the present results and previous studies^13,34–39^ have indicated that PMOA is always associated with increases in oxidative stress, cytoplasmic calcium, activation susceptibility, spindle/chromosome abnormalities and cytoplasmic fragmentation and with impairment of developmental potential, mitochondrial function and CG distribution. The current results showed that upregulating autophagic activities with rapamycin or LiCl inhibited PMOA by correcting these aging parameters, whereas down regulating autophagy with 3-MA accelerated PMOA by aggravating the aging symptoms. It is known that the activation susceptibility of aging oocytes negatively correlates with levels of maturation-promoting factor (MPF) and mitogen-activated protein kinase (MAPK) activities^6,40^, and that activated protein kinase A maintains both MPF and MAPK in an inactive state^41,42^. Inhibition of TOR with rapamycin leads to PKA inactivation, and genes involved in almost all steps of autophagy were detected as candidates for PKA negative regulators^43^. Thus, treatment with rapamycin might have reduced oocyte activation susceptibility by activating MPF and MAPK via inhibiting PKA.

Studies in somatic cells have shown that Ca^2+^ homeostasis perturbation is the major factor influencing autophagy^44^. ROS are known to significantly perturb Ca^2+^ homeostasis through direct effects on intracellular Ca^2+^ storage^45,46^ and/or on the Na^+^/Ca^2+^ exchanger activity^38^. Furthermore, increasing ROS levels induces lysosomal Ca^2+^ release, which enhances autophagy by triggering PPP3/calcineurin-dependent transcription factor EB nuclear translocation^47^. On the other hand, autophagy is one of the first lines of defense against oxidative stress damage^48,49^. There are reports that highlight the role of autophagy as a critical protective mechanism against mitochondrial dysfunction. In human cells, accumulation of ROS led to changes in mitochondrial permeability with loss of MMP and disruption of mitochondrial dynamics^50^. Treatment of rat heart with anti-β1-adrenergic receptor antibodies significantly decreased MMP potential with a marked decrease in autophagy^51^. Furthermore, autophagy activation with rapamycin significantly protected against mitochondrial dysfunction, whereas genetic inhibition of autophagy induced significant mitochondrial function defects in human chondrocytes^52^.

In summary, we propose that autophagy plays an important role in regulating PMOA. During the early stage of PMOA, autophagy increases as an adaptive response to prevent further apoptosis, but by the late stage of PMOA, the activation of more caspases blocks the autophagic process leading to severer apoptosis. We believe that our data are important for understanding the mechanisms not only for oocyte aging but also for autophagy and the crosstalk between autophagy and apoptosis.

## Materials and methods

All the procedures for animal care and handling were approved by the Animal Care and Use Committee of the Shandong Agricultural University P. R. China (Permit number: SDAUA-2001-001). All the chemicals and reagents used were purchased from Sigma Chemical Company unless otherwise mentioned.

### Oocyte collection

Mice of Kunming breed were raised in a room with 14 h light: 10 h dark cycles, with the dark period starting from 8 pm. Female mice, at the age of 8–10 weeks, were induced to superovulate with 10 IU equine chorionic gonadotropin (eCG, i.p.), followed 48 h later by 10 IU human chorionic gonadotropin (hCG, i.p.). Both eCG and hCG were purchased from Ningbo Hormone Product company limited, China. The superovulated mice were sacrificed 13 h after hCG administration to recover oocytes by breaking the oviductal ampullae. After being washed three times in M2 medium^53^, the oocytes were denuded of cumulus cells by pipetting in M2 containing 0.1% hyaluronidase and the cumulus-free oocytes were used for experiments.

### Preparation of FCM

A FCM was prepared as reported previously^33^. The cumulus cells released during preparation of cumulus-free oocytes were harvested and cultured for 24 h in regular CZB medium^54^ containing 200 µM H_2_O_2_. The H_2_O_2_-treated cumulus cells were then incubated in regular CZB for 48 h, and at the end of the incubation, the conditioned medium was recovered and centrifuged at 3000×*g* for 5 min to remove cells and debris. The FCM obtained was frozen at −80 °C until use.

### Oocyte aging in vitro

Cumulus-free oocytes were cultured for aging in regular CZB medium, FCM or FCM containing various concentrations of MG132, rapamycin, LiCl or 3-MA. To prepare stock solutions, MG132 (5 mM) and rapamycin (10 µM) were dissolved in dimethyl sulfoxide (DMSO), whereas 3-MA (10 mM) and LiCl (10 M) were dissolved in CZB medium and PBS, respectively. All the stock solutions were stored in aliquots at −20 °C and they were diluted to desired concentrations with corresponding aging media immediately before use. The aging culture was performed in wells (about 30 oocytes per well) of a 96-well culture plate containing 100 µl of medium at 37 °C under 5% CO_2_ in humidified air.

### Oocyte activation

Two activation methods were adopted in this study: ethanol plus 6-dimethylaminopurine (6-DMAP) was used to assess oocyte activation susceptibility, and the SrCl_2_ was used to evaluate oocyte developmental potential. During ethanol + 6-DMAP treatment, oocytes were first treated with 5% ethanol in M2 medium for 5 min at room temperature, and then cultured in 2 mM 6-DMAP in CZB medium for 6 h at 37.5 °C in a humidified atmosphere containing 5% CO_2_ in air. For SrCl_2_ activation, oocytes were incubated for 6 h in Ca^2+^-free CZB medium supplemented with 10 mM SrCl_2_ and 5 µg/ml cytochalasin B. At the end of the activation culture, oocytes were observed under a inverted microscope for activation. Oocytes showing one or two pronuclei, or showing two cells each having a nucleolus, were judged as activated.

### Embryo culture

The Sr^2+^-activated oocytes were cultured in regular CZB medium (about 30 oocytes per well containing 100 µl medium) at 37.5 °C under a humidified atmosphere with 5% CO_2_ in air. On day 2 of culture, oocytes were examined for four-cell development, and were transferred to CZB medium containing 5.55 mM glucose for further culture. On day 4.5 of the culture, oocytes were examined for blastocyst formation.

### Western blot analysis

Two to three hundreds (for LC3 and p62) or 300–400 (for active caspase-3) cumulus-free oocytes were frozen at −80 °C in a 1.5-ml microfuge tube containing 20 µl sample buffer (20 mM Hepes, 100 mM KCl, 5 mM MgCl_2_, 2 mM dithiothreitol, 0.3 mM phenylmethyl sulfonyl fluoride, 3 mg/ml leupetin, pH 7.5). To extract protein, 6.67 µl of 5× SDS-PAGE loading buffer was added to each tube and the tubes were heated for 5 min at 100 °C. A SDS-PAGE was performed on a 15% polyacrylamide gel to separate total proteins, and the proteins obtained were transferred onto polyvinylidene fluoride membranes via electrophoresis. The membranes were then (1) washed three times in TBST (50 mM NaCl, 2 mM KCl, 25 mM Tris, 0.05% Tween-20, pH 7.4); (2) blocked for 1–1.5 h at room temperature with TBST containing 3% BSA; (3) incubated overnight at 4 °C with primary antibodies (1: 1000) in 3% BSA-TBST; (4) washed three times (10 min each) in TBST and incubated for 1 h at 37 °C with second antibodies (1: 1000) in 3% BSA-TBST; (5) washed three times in TBST and detected using a BCIP/NBT alkaline phosphatase color development kit (Beyotime Institute of Biotechnology, China). For internal controls, β-tubulin or GAPDH was also assayed. To determine the relative quantities of proteins, the sum density of each protein band image was analyzed using an Image-Pro Plus software. Ratios of target proteins/internal control (β-tubulin or GAPDH) were calculated to represent the quantity values of each target proteins. The primary antibodies used included rabbit anti-LC3 antibody (4108, Cell Signaling, USA), rabbit anti-active caspase-3 polyclonal antibodies (ab13847, Abcam Co. Ltd, Cambridge, MA, USA), rabbit anti-P62 antibody (5114 S, Cell Signaling), mouse anti-β-tubulin monoclonal antibodies (05-661, Merck Millipore, China), and mouse anti-GAPDH monoclonal antibody (CW0100A, CWBIO, China). The secondary antibodies included goat anti-rabbit IgG AP conjugated (cw0111, CWBIO) and goat anti-mouse IgG AP conjugated (cw0110, CWBIO).

### Cytoplasmic calcium measurement

To load the Ca^2+^ probe, cumulus-denuded oocytes were incubated at room temperature for 30 min in HCZB medium containing 1 µM Fura-2 AM and 0.02% pluronic F-27. Drops of HCZB medium were made in a Fluoro dish (FD35-100, World Precision Instruments) and covered with mineral oil. Oocytes were transferred into the HCZB drops and observed with a Leica DMI6000 inverted microscope at 37 °C. A Fura 2 fluorescence module was used for excitation, and a Leica LAS-AF calcium imaging module was used to calculate the F340/380 ratio. The oocytes were monitored for 10 min to record the F340/380 ratio, which represented the concentration of cytoplasmic calcium.

### Measurement of intra-oocyte reactive oxygen species (ROS)

Intra-oocyte ROS was quantified by measuring H_2_O_2_ levels using 2′,7′-dichlorodihydro-fluorescein diacetate (DCHFDA). Stock solution of DCHFDA (1 mM) was made by dissolving DCHFDA in DMSO and was stored in the dark at −20 °C. Immediately before use, the stock solution was diluted to 10 µM with M2 medium, and cumulus-denuded oocytes were stained with the resultant solution at 37 °C for 10 min. At the end of the staining, the oocytes were thoroughly washed in M2, placed on a slide, and examined under a Leica confocal microscope (TCS SP2) with fluorescence obtained by excitation at 488 nm. All the photographs were taken using fixed microscopic parameters. The fluorescence intensity of each oocyte was analyzed using a Leica software.

### Measurement of MMP

An MMP detection (JC-1) kit (Beyotime Biotechnology Research Institute, China) was used to detect MMP. Briefly, cumulus-free oocytes were washed three times with M2 and placed in a drop of 1 ml M2 and 1 ml JC-1 dye working solution. The oocytes were then incubated at 37 °C for 25 min. After being washed three times with a JC-1 staining buffer, the oocytes were observed under a Leica laser scanning confocal microscope. The same oocytes were observed through TRITC channel (red fluorescence) and FITC channel (green fluorescence). The aggregate JC-1 (red fluorescence) was detected at an emission wave length of 590 nm, while the monomeric JC-1 (green fluorescence) was monitored at 529 nm. The ratio of aggregated/ monomeric JC-1 was calculated to quantify changes in MMP, with a decreased red/green JC-1 ratio representing depolarization of the mitochondria.

### Immunofluorescence microscopy for detection of LC3, active caspase-3, CGs and tubulin

Procedures were performed at room temperatures unless otherwise specified. Cumulus-free oocytes were washed three times in M2 between treatments. Oocytes were (1) fixed for 30 min with 3.7% paraformaldehyde contained in PBS; (2) treated with 0.5% protease in M2 for 10 seconds to remove zona pellucida; (3) permeabilized at 37.5 °C for 10 min in PBS containing 0.1% Triton X-100; and (4) blocked with 3% BSA in PBS at 37.5 °C for 0.5 h. To detect LC3, blocked oocytes were incubated at 4 °C overnight with rabbit anti-LC3 antibody (1:300, 4108, Cell Signaling) in the antibody diluent, and then, incubated for 1.5 h in the dark with Cy3-conjugated goat-anti-rabbit IgM (1:100, 111-165-144, Jackson ImmunoResearch, USA), and finally, oocytes were incubated for 5 min with 10 µg/ml Hoechst 33342 in M2 to stain chromatin. For active caspase-3 detection, the blocked oocytes were incubated at 37.5 °C for 1 h first with mouse anti-active-caspase-3 (IgM, 1:100, Millipore) in 1% BSA in PBS, and then, with FITC-conjugated donkey-anti-mouse IgM (1:200, 715-095-020, Jackson ImmunoResearch) in 3% BSA in PBS. Then, the oocytes were incubated for 5 min with 10 µg/ml Hoechst 33342 in M2 to stain chromatin. Samples in which the primary antibody was omitted were also processed to serve as negative controls. For tubulin staining, the blocked oocytes were first incubated at 37 °C for 1 h in PBS containing FITC-conjugated anti-α-tubulin monoclonal antibodies (1:50), and then, incubated for 5 min with 10 µg/ml Hoechst 33342 in M2 to stain chromatin. To stain CGs, the blocked oocytes were incubated for 1 h in 100 µg/ml of FITC-labeled lens culinaris agglutinin in M2 followed by incubation for 5 min in M2 containing 10 µg/ml Hoechst 33342 to stain chromatin. The stained oocytes were examined under a Leica confocal microscope, and blue diode (405 nm), argon (488 nm) and helium/neon (He/Ne; 543 nm) lasers were used to excite Hoechst, FITC and Cy3, respectively. Fluorescence was detected with 420-480 nm (Hoechst), 492–520 nm (FITC) and 550–570 nm (Cy3) filters, and the captured signals were recorded as blue, green and red, respectively. To quantify the active caspase-3 expression, the relative fluorescence intensities were measured on the raw images using Image-Pro Plus software (Media Cybernetics Inc., Silver Spring, MD) under fixed thresholds across all slides.

### Data analysis

In all the experiments, each treatment was repeated at least three times. Data were arc sine transformed before being analyzed with ANOVA. A Duncan multiple test was performed to determine the differences. The software of SPSS 11.5 (SPSS Inc.) was used. Data are expressed as mean ± SEM, and *P* < 0.05 was considered significant.
